# The Explosion on January 19

**Published:** 1917-02-03

**Authors:** C. E. Leo Lyle

**Affiliations:** Chairman, Queen Mary's Hospital for the East End, Stratford, E.


*To the Editor of* The Hospital.

### The Explosion on January 19.

*To the Editor of* The Hospital.

Sir,—I have read many accounts of the explosion and the events immediately following upon it in various journals, but in one only have I seen the name of Queen Mary's Hospital for the East End mentioned in connection with the work of succouring the poor unfortunate people who were involved.

I am inclined to think that this may be due to the idiosyncrasy of that much criticised individual, the Censor, as I have seen the hospital referred to as "another hospital" in connection with the local coroner's inquest and in other connections.

But since both the London Hospital and Poplar Hospital are mentioned by name in a long account in your issue of the 27th inst., referring to the splendid work performed by them on that terrible night, I feel it is only right to let it be known that Queen Mary's Hospital for the East End also took a prominent part in tending those who suffered in that dreadful catastrophe.

Queen Mary's Hospital for the East End treated 110 victims of the explosion, of whom twenty-five remained as in-patients.—I am, Sir, yours truly,

C. E. Leo Lyle,
Chairman, Queen Mary's Hospital for the East End, Stratford, E.

January 29, 1917.

